# Chronic tooth pulp inflammation induces persistent expression of phosphorylated ERK (pERK) and phosphorylated p38 (pp38) in trigeminal subnucleus caudalis

**DOI:** 10.1016/j.neuroscience.2014.03.056

**Published:** 2014-06-06

**Authors:** M.A. Worsley, C.E. Allen, A. Billinton, A.E. King, F.M. Boissonade

**Affiliations:** a Unit of Oral & Maxillofacial Medicine & Surgery, School of Clinical Dentistry, University of Sheffield, Claremont Crescent, Sheffield S10 2TA, UK; b Neuroscience, GlaxoSmithKline, Harlow, UK; c School of Biomedical Sciences, University of Leeds, UK

**Keywords:** ANOVA, analysis of variance, AOI, area of interest, CFA, complete Freund’s adjuvant, Cy3, indocarbocyanine, EMG, electromyogram, ERK, extracellular signal-regulated kinase, FITC, fluorescein isothiocyanate, GFAP, glial fibrillary acidic protein, JOR, Jaw-opening reflex, MAPK, mitogen-activated protein kinase, NDS, normal donkey serum, NeuN, neuron-specific nuclear protein, NGS, normal goat serum, p38, p38 MAPK, PBS, phosphate-buffered saline, PBST, phosphate-buffered saline containing Triton-X, pERK, phosphorylated ERK, pp38, phosphorylated p38, Vc, trigeminal subnucleus caudalis, pERK, pp38, MAPK, chronic inflammation, pain, trigeminal nucleus

## Abstract

**Background:**

Extracellular signal-regulated kinase (ERK) and p38 mitogen-activated protein kinase are transiently phosphorylated (activated) in the spinal cord and trigeminal nucleus by acute noxious stimuli. Acute stimulation of dental pulp induces short-lived ERK activation in trigeminal subnucleus caudalis (Vc), and p38 inhibition attenuates short-term sensitization in Vc induced by acute pulpal stimulation. We have developed a model to study central changes following chronic inflammation of dental pulp that induces long-term sensitization. Here, we examine the effects of chronic inflammation and acute stimulation on the expression of phosphorylated ERK (pERK), phosphorylated p38 (pp38) and Fos in Vc.

**Results:**

Chronic inflammation alone induced bilateral expression of pERK and pp38 in Vc, but did not induce Fos expression. Stimulation of both non-inflamed and inflamed pulps significantly increased pERK and pp38 bilaterally; expression was greatest in inflamed, stimulated animals, and was similar following 10-min and 60-min stimulation. Stimulation for 60 min, but not 10 min, induced Fos in ipsilateral Vc; Fos expression was significantly greater in inflamed, stimulated animals. pERK was present in both neurons and astrocytes; pp38 was present in neurons and other non-neuronal, non-astrocytic cell types.

**Conclusions:**

This study provides the first demonstration that chronic inflammation of tooth pulp induces persistent bilateral activation of ERK and p38 within Vc, and that this activation is further increased by acute stimulation. This altered activity in intracellular signaling is likely to be linked to the sensitization that is seen in our animal model and in patients with pulpitis. Our data indicate that pERK and pp38 are more accurate markers of central change than Fos expression. In our model, localization of pERK and pp38 within specific cell types differs from that seen following acute stimulation. This may indicate specific roles for different cell types in the induction and maintenance of pulpitic and other types of pain.

## Highlights

• Chronic inflammation of tooth pulp activates pERK and pp38 in the trigeminal nucleus
• Activation is persistent and bilateral, and further increased by acute stimulation
• This altered signaling may be relevant in the development of chronic pulpitic pain
• pERK and pp38 are more sensitive markers of central change than Fos expression
• Sequential activation in different cell types may be linked to pain progression

## Introduction

Evidence indicates that altered activity in intracellular signaling cascades plays a significant role in altered excitability and sensitization in the spinal cord and trigeminal nucleus, and this activity has been implicated in the development of pain. The mitogen-activated protein kinases (MAPKs) extracellular signal-regulated kinase (ERK) and p38 MAPK are phosphorylated (activated) in the spinal cord and trigeminal nucleus by noxious stimulation. It is also well established that the proto-oncogene c-fos and its protein product Fos can be rapidly induced in these regions by peripheral noxious stimulation. Thus, these molecules have been widely used as correlates of activity related to nociception.

Although Fos, phosphorylated ERK (pERK) and phosphorylated p38 (pp38) are induced by a range of peripheral stimuli, the time course of expression of these molecules varies widely according to the stimulus. For example a number of studies have described relatively short-term increases (peak 2–20 min, returning to baseline levels at ∼2 h) in levels of pERK following the application of a number of acute noxious stimuli including electrical, thermal, mechanical, and chemical stimulation (mustard oil, capsaicin, and carrageenan) (Ji et al., 1999, 2002; Galan et al., 2002; Pezet et al., 2002). A study in the trigeminal system (Huang et al., 2000) describes increased numbers of pERK-immunoreactive (IR) neurons in ipsilateral trigeminal subnucleus caudalis (Vc) 1 h after formalin injection into the peri-oral skin. However, some studies in models of chronic inflammation have found that increased pERK levels are maintained for long periods under these conditions. For example, increased levels of pERK still persist 7 days after injection of complete Freund’s adjuvant (CFA) into the hind paw (Adwanikar et al., 2004), whereas a study in a neuritis model (Kominato et al., 2003) demonstrated that pERK (and Fos) was not increased in animals with neuritis alone but, following noxious stimulation (pinch) 3 and 7 days after induction of neuritis, levels of pERK (and Fos) were greater in animals with neuritis than in control animals.

The MAPK p38 is also activated by a variety of acute stimuli including injection of intraplantar formalin and intrathecal substance P (Svensson et al., 2003). This increase in activity is short lived (present at 5 min, returning to baseline by 20 min). More persistent activation of p38 has also been reported in other models, for example ligation of L5 spinal nerve results in activation of p38 starting within 1 day of the lesion which persists for longer than 3 weeks (Jin et al., 2003). Unlike the increases in pERK, p38 activation has generally been reported in glia rather than neurons (Ji et al., 2009). This increase in p38 activity has also been linked to neuronal Fos expression as p38 MAPK inhibition prevented Fos expression (and attenuated hyperalgesia) following intraplantar formalin injection (Svensson et al., 2003).

We have developed a model to study central changes following chronic inflammation of the dental pulp in the ferret and have examined Fos expression in the trigeminal nucleus following electrical tooth pulp stimulation in animals with normal and inflamed tooth pulps (Oakden and Boissonade, 1998; Worsley et al., 2007, 2008). In this model, unlike that reported in models of acute pulpal inflammation (e.g. stimulation with lipopolysaccharide (Chattipakorn et al., 2002)), we do not see Fos protein expressed under conditions of inflammation alone. However when the inflamed pulp is electrically stimulated 5 days post induction of inflammation, Fos expression is significantly greater than that seen following similar stimulation of the non-inflamed pulp (Worsley et al., 2007, 2008). This indicates that in this model although the inflammation alone does not induce Fos expression, it does induce long-term sensitization. Previous studies have shown that acute stimulation of the dental pulp induces short-lived ERK activation in the trigeminal nucleus (Shimizu et al., 2006), but this was shown to return to baseline levels 2 h post stimulus. It has also been demonstrated that inhibition of p38 can attenuate central sensitization in Vc nociceptive neurons induced by acute noxious stimulation of the tooth pulp with mustard oil (Xie et al., 2007). However it is not yet established whether these molecules are activated for longer time periods in chronic pulpal inflammation, such as that which occurs in patients with pulpal inflammatory pain.

Persistent activation of these molecules in our model of chronic pulpal inflammation could contribute to the increased Fos expression seen following stimulation of inflamed tooth pulp. Thus our model provides an excellent opportunity to identify central changes in MAPK activation that may play a role in central sensitization within the trigeminal system.

The aim of this study was to determine the effect of chronic inflammation on expression of pERK, pp38 and Fos. We compared expression of pERK, pp38 and Fos in animals with non-inflamed and chronically inflamed pulps, and examined their expression following electrical stimulation (for 10 or 60 min) of the tooth pulp in both groups of animals. In some sections, we carried out dual labeling to examine colocalization of pERK and pp38 with the neuronal marker neuron-specific nuclear protein (NeuN) and the astrocyte marker glial fibrillary acidic protein (GFAP).

This paper reports that chronic inflammation of the tooth pulp induces significant and persistent bilateral expression of pERK and pp38 in Vc. The pERK is present in neurons and astrocytes, while the pp38 is seen in neurons and other non-neuronal, non-astrocytic cell types.

## Experimental procedures

Experiments were carried out in 32 adult female ferrets (5–8 months old; Highgate Farm, UK), under UK Home Office Licence regulation and approval. The ferrets were prepared under anesthesia (ketamine, Fort Dodge, Southampton, UK; 25 mg/kg; xylazine, Bayer, Newbury, UK; 2 mg/kg; intramuscularly) in order to allow tooth pulp inflammation and stimulation of the upper and lower left canine teeth, and an electromyogram (EMG) to be recorded from the digastric muscle during a subsequent experiment. Briefly, a connector block composed of a mini 9-way socket and an intravenous injection port was attached to the skull. Leads from this block were passed subcutaneously to insulated Ag–AgCl fillings in the left canine teeth for stimulation, and to the left digastric muscle for recording its EMG. A cannula from the injection port was inserted into the left jugular vein. Following cannula insertion anesthesia was maintained intravenously, using alfaxalone (Vétoquinol; Buckingham, UK) at 6 mg/kg/h. These procedures have been described in greater detail previously (Oakden and Boissonade, 1998; Worsley et al., 2007). In 16 of these animals, pulpal inflammation was induced by the introduction of human caries into deep buccal cavities in the upper and lower left canine teeth as described previously (Worsley et al., 2007).

Five days later the animals were anesthetized with alfaxalone (4 mg/kg) via the indwelling cannula. Light anesthesia was then maintained by continuous infusion of alfaxalone (4–14 mg/kg/h), adjusted to allow a withdrawal reflex to be seen following a paw squeeze. Body temperature was maintained at 38 ± 0.5 °C. In 16 animals (non-inflamed *n* = 8, inflamed *n* = 8) the tooth pulps were electrically stimulated via the tooth electrodes (a train of three 0.5-ms duration stimuli at 200 Hz) and the amplitude of the stimulus required to produce the jaw-opening reflex (JOR) was determined for each tooth. The tooth pulps were then stimulated at ten times the JOR threshold, once per second for either 10 min (non-inflamed *n* = 4, inflamed *n* = 4) or 60 min (non-inflamed *n* = 4, inflamed *n* = 4). The remaining 16 animals were kept under anesthesia for either 10 min (non-inflamed *n* = 4, inflamed *n* = 4) or 60 min (non-inflamed *n* = 4, inflamed *n* = 4). All animals were then immediately deeply anesthetized with sodium pentobarbitone (Sagatal; Rhone Merieux, Harlow, UK; 42 mg/kg, intraperitoneally) and perfused with 1000 ml of phosphate-buffered saline (PBS), followed by 1000 ml of 4% paraformaldehyde fixative.

Following perfusion brainstems were removed, post-fixed in paraformaldehyde for 4 h at 4 °C and immersed in a 30% sucrose solution overnight for cryoprotection.

### Immunohistochemistry

Following overnight immersion in sucrose solution the brainstems were marked along the ventral surface to allow identification of the left and right sides. They were then embedded in optimum cutting temperature medium (OCT; Tissue Tek, UK) and stored at −80 °C until cut. Frozen serial sections (30 μm) were cut from 5 mm caudal to 10 mm rostral to obex (the point at which the central canal opens up into the fourth ventricle). Sections were collected in four sets, generating 120 μm between adjacent sections in each set. Sets 1–3 were processed immunohistochemically for Fos, pERK and pp38. The fourth set was used for double labeling of pERK or pp38, with either the neuronal marker NeuN or the astrocyte marker GFAP.

#### Single labeling of Fos, pERK and pp38

The first set of tissue was processed for Fos immunohistochemistry, as described previously (Worsley et al., 2007). The second and third sets were processed for pERK and pp38, respectively. In order to reduce background staining the sections were placed in 10% normal goat serum (NGS; Vector Laboratories, Peterborough, UK) made up in PBS containing 0.2% Triton X-100 (PBST) for 1 h. They were then incubated at 4 °C for 48 h with primary antibodies raised in rabbit to one of: Fos (1:20,000; Calbiochem, San Diego, CA, USA); pERK (1:600 [#9101]; Cell Signaling Technology, Danvers, MA, USA), or pp38 (1:300 [#9211]; Cell Signaling Technology) (see Table 1). In all cases, the primary antibodies were made up in PBST containing 5% NGS. The sections were then washed twice with PBS and incubated for 30 min at room temperature with a biotinylated anti-rabbit antibody raised in goat (1:300; Vector Laboratories, UK), made up in PBST containing 1.5% NGS. The labeling was visualized using the avidin–biotin complex (ABC) method with nickel-intensified diaminobenzidine (DAB) as the chromagen, as described previously (Worsley et al., 2007). Immunohistochemical controls were performed using liquid phase pre-absorption of the relevant primary antibody with the Fos peptide (Calbiochem), pERK peptide (#1150; Signaling Technology) or pp38 peptide (#1170; Cell Signaling Technology).

#### Double labeling

Sections from the fourth set of tissue were used for dual-labeling of pERK or pp38, with either NeuN or GFAP to identify cell types expressing pERK or pp38.

In order to reduce background staining, the sections were first placed in 10% normal donkey serum (NDS; Vector Laboratories, UK) made up in PBST for 1 h. Sections were then incubated for 24 h with primary antibodies raised in rabbit to one of pERK or pp38 (as above and at the same dilutions as for the single labeling studies), together with mouse monoclonal primary antibodies to either NeuN (1:500; Abcam, Cambridge, UK) or GFAP (1:300; Vector Laboratories, UK) (Table 1). Primary antibodies were diluted in PBST containing 5% NDS.

After washing with PBS, sections were incubated for 2 h with indocarbocyanine (Cy3)-labeled donkey anti-rabbit, together with fluorescein isothiocyanate (FITC)-labeled donkey anti-mouse secondary antibodies (both 1:500; Jackson ImmunoResearch, Newmarket, UK), diluted in PBST containing 2% NDS. Thus pERK and pp38 labeling were visualized with Cy3, and NeuN and GFAP labeling were visualized with FITC.

Negative controls for the monoclonal antibodies to NeuN and GFAP were performed where the primary antibodies were omitted and the tissue incubated with NDS and the FITC-labeled secondary antibody only.

### Analysis

Sections were viewed using a Zeiss Axioplan 2 microscope equipped for fluorescence imaging, and Image Pro-Plus (v5.1, Media Cybernetics, Rockville, MD, USA) was used for image capture and analysis. Fos, pERK and pp38 immunohistochemistry was examined in seven sections at 120-μm intervals from 1800 μm caudal to 1080 μm caudal to obex. All slides were blinded prior to experimental analysis, and all quantification was undertaken by the same investigator and repeated on 10% of samples on a different day. These repeat measurements were within a 5% margin of the first count.

#### Fos

The Fos-positive nuclei in Vc, on both the contralateral (unstimulated, right) and ipsilateral (stimulated, left) sides of the brainstem were counted at the seven levels described above. Statistical comparisons of total Fos counts between treatment groups were made using the unpaired Student’s *t*-test.

#### pERK and pp38

Computerized image analysis of labeling was carried out using Image-Pro Plus v5.1 computer software (Media Cybernetics, USA) to quantify the percentage area of labeling for each of the molecules. We used an area of interest (AOI) tool to generate a series of areas that outlined the region in the outer laminae of Vc where Fos was expressed following pulpal stimulation (examples of these outlines are shown in Fig. 2). Outlines were made for each of the seven levels where Fos expression was quantified. These AOIs were superimposed onto captured images of pERK and pp38 labeling from equivalent rostro–caudal regions of Vc. The percentage area of the AOI that contained labeling for pERK and pp38 was calculated and an average of the seven levels used as the value for each animal.

#### Statistics

Statistical comparison of areas expressing pERK and pp38 were made between different treatment groups using a two-way analysis of variance (ANOVA). These comparisons demonstrated a highly significant effect of inflammation, thus the unpaired Student’s *t*-test was used to compare non-stimulated, non-inflamed vs. non-stimulated, inflamed groups; and stimulated, non-inflamed vs. stimulated, inflamed groups.

As Fos was only expressed in the groups stimulated for 60 min, statistical comparisons of Fos counts were made between the 60-min stimulated, non-inflamed and 60-min stimulated, inflamed groups using the unpaired Student’s *t*-test.

A value of *p* < 0.05 was considered significant in all statistical tests.

## Results

**Fig. 1:**
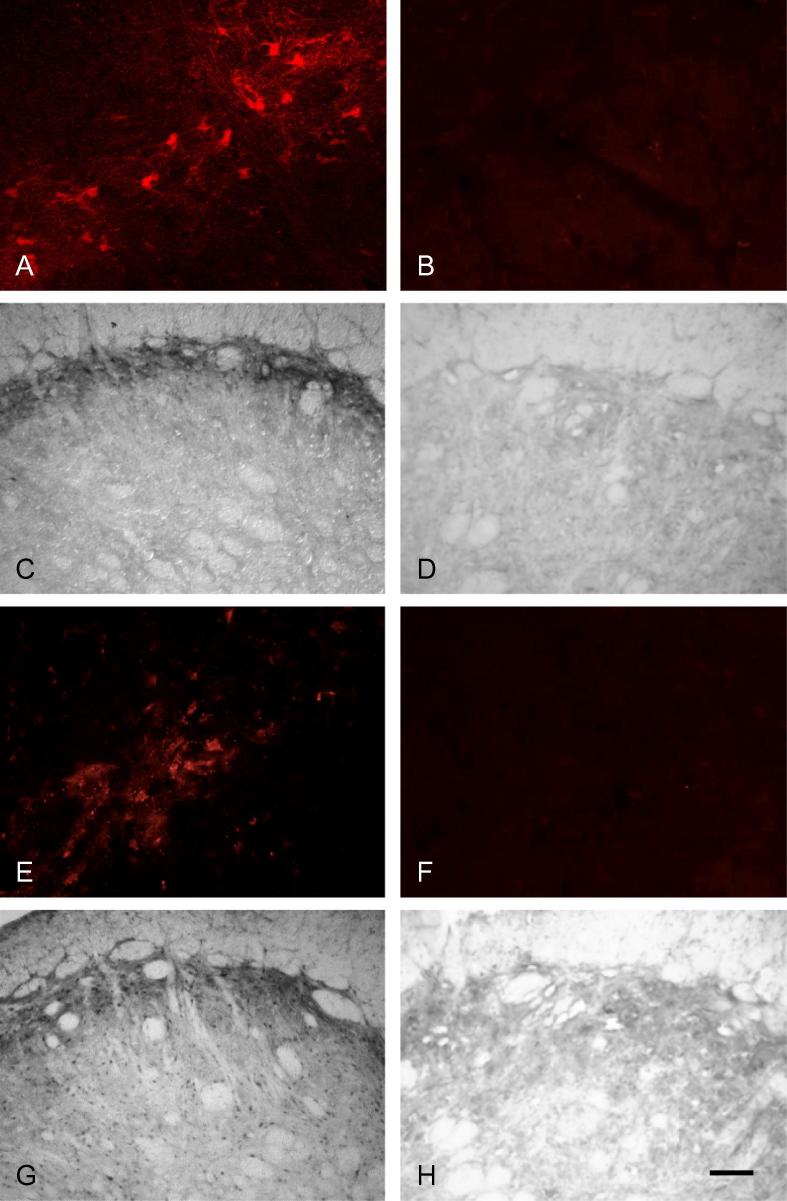
pERK and pp38 antibodies: preabsorption controls. Photomicrographs illustrating (A–D) pERK and (E–H) pp38 immunohistochemistry in subnucleus caudalis. Preabsorption of the pERK and pp38 antibodies with the respective peptides abolished immunoreactivity (B, D, F, H). Scale bar = 100 μm (C, D, G, H); 50 μm (A, B, E, F).

pERK and pp38 immunoreactivity was present in subnucleus caudalis in all groups and was abolished by preabsorption of the antibodies with their respective peptides (Fig. 1). Preabsorption of the Fos antibody with its respective peptide also abolished Fos immunoreactivity, as described previously (Bowler et al., 2013).

### Fos labeling

**Fig. 2:**
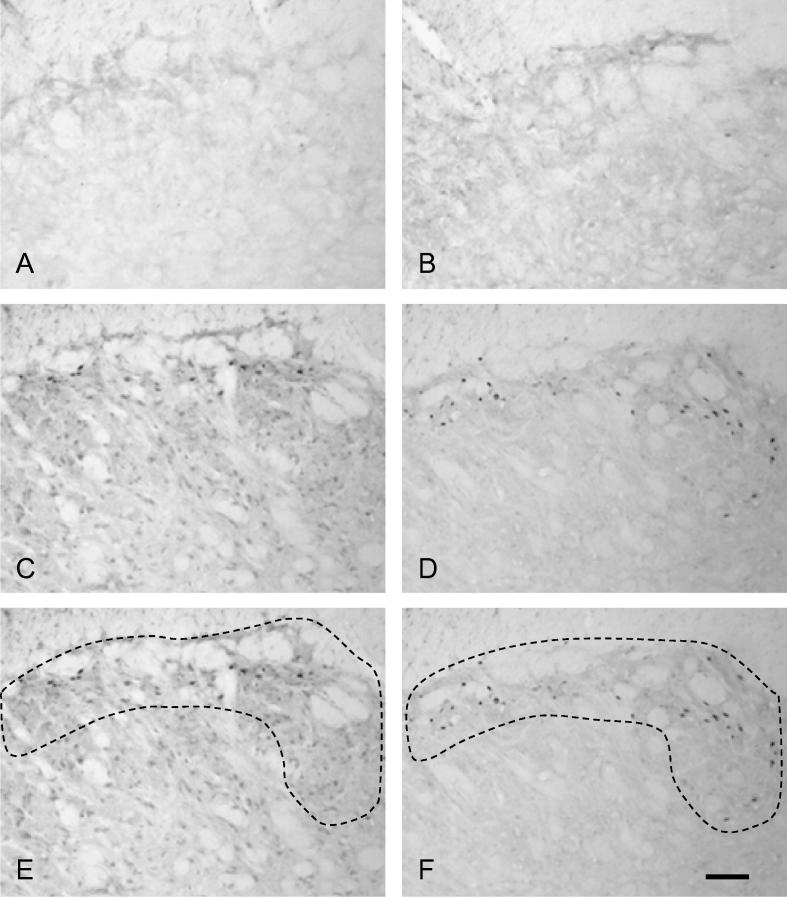
Fos immunohistochemistry in subnucleus caudalis from animals with stimulated and/or inflamed tooth pulps. Photomicrographs showing Fos-positive staining in ipsilateral subnucleus caudalis (1800 μm caudal to obex). (A) non-stimulated, non-inflamed; (B) non-stimulated, inflamed; (C) stimulated (60 min), non-inflamed; (D) stimulated (60 min), inflamed; (E) and (F) images from (C) and (D) outlining regions where Fos-positive cells were counted. Scale bar = 100 μm.

Fos labeling was only present in the trigeminal nucleus of the two groups of animals that had received pulpal stimulation for 60 min. As expected, no Fos was present in the groups of animals that were perfused after 10 min of pulpal stimulation; this is in keeping with previous studies showing that Fos first appears ∼20 min after the application of a stimulus. As described in our previous study (Worsley et al., 2007) there was also very little Fos in non-stimulated animals with either normal or inflamed pulps (Fig. 2). When present, Fos-positive profiles were concentrated on the left side (ipsilateral to pulpal stimulation) of the trigeminal nucleus with very little Fos labeling in the contralateral brainstem, this is also as described in our previous studies (Oakden and Boissonade, 1998; Worsley et al., 2007, 2008; Bowler et al., 2013).

In subnucleus caudalis, Fos expression was seen in the outer laminae, between 2730 and 930 μm caudal to obex, peaking at approximately 1530 μm. These findings are in agreement with previously published results and are described in more detail elsewhere (Oakden and Boissonade, 1998; Worsley et al., 2007, 2008; Bowler et al., 2013).

**Fig. 3:**
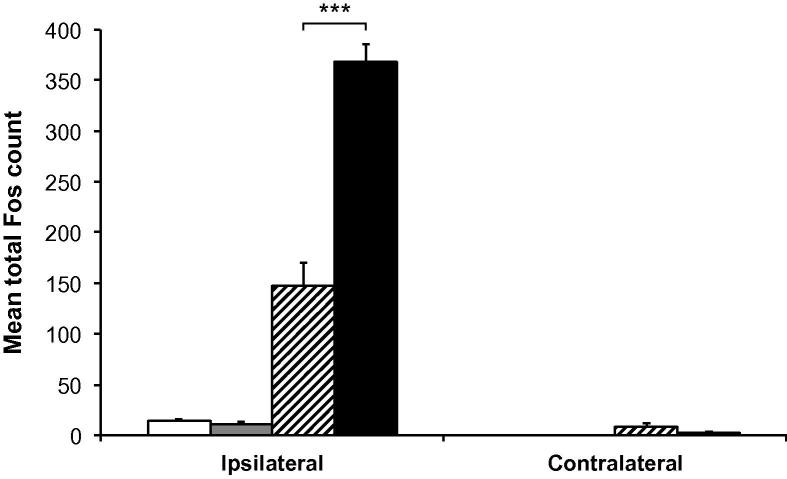
Mean total Fos counts in subnucleus caudalis. Mean total Fos count in both ipsilateral and contralateral subnucleus caudalis of animals with non-inflamed or inflamed tooth pulps, either non-stimulated or stimulated for 60 min. □ = non-stimulated, non-inflamed;  = non-stimulated, inflamed;  = stimulated, non-inflamed; ■ = stimulated, inflamed. Each bar represents the average total Fos count of seven sections in the area 1800–1080 μm caudal to obex. ^∗∗∗^*p* < 0.001; unpaired *t*-test.

Following 60 min of stimulation, Fos-positive profiles appeared more numerous in animals with inflamed pulps than in those with non-inflamed pulps (Fig. 2C, D). Quantification of these profiles revealed that total Fos expression in Vc was significantly greater in animals with inflamed pulps (mean 368.0 ± 17.8 [SEM] cells) than in those with non-inflamed pulps (mean 147.8 ± 22.6 [SEM] cells; *p* = 0.0003, unpaired *t*-test; Fig. 3).

### pERK and pp38 labeling

**Fig. 4:**
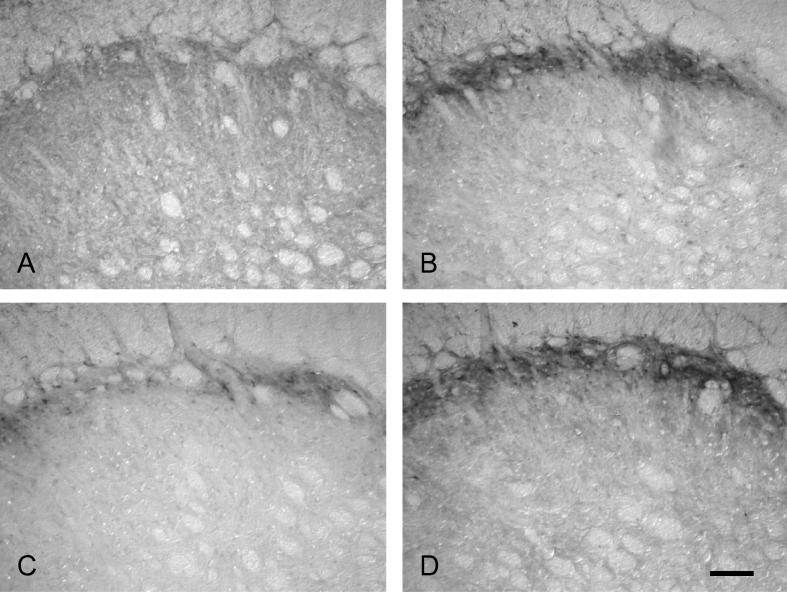
Examples of pERK immunoreactivity in subnucleus caudalis. Photomicrographs showing examples of ipsilateral pERK labeling in subnucleus caudalis (1800 μm caudal to obex). (A) non-stimulated, non-inflamed; (B) non-stimulated, inflamed; (C) stimulated (10 min), non-inflamed; (D) stimulated (10 min), inflamed. Scale bar = 100 μm.

Labeling for pERK and pp38 appeared similar on both right and left sides of the brainstem (i.e., ipsilateral and contralateral to the prepared teeth). Expression of pERK and pp38 was concentrated in the outer laminae (Figs. 4 and 6) both ipsilaterally and contralaterally, in a similar region to that where Fos expression was seen ipsilaterally. Labeling was present in profiles representing both cell bodies and processes (Fig. 1). Expression of pERK and pp38 was very low in non-stimulated animals with non-inflamed pulps, and levels of expression appeared highest in stimulated animals with inflamed pulps. There appeared to be little difference in levels of pERK and pp38 between animals receiving either 10 or 60 min of stimulation.

**Fig. 5:**
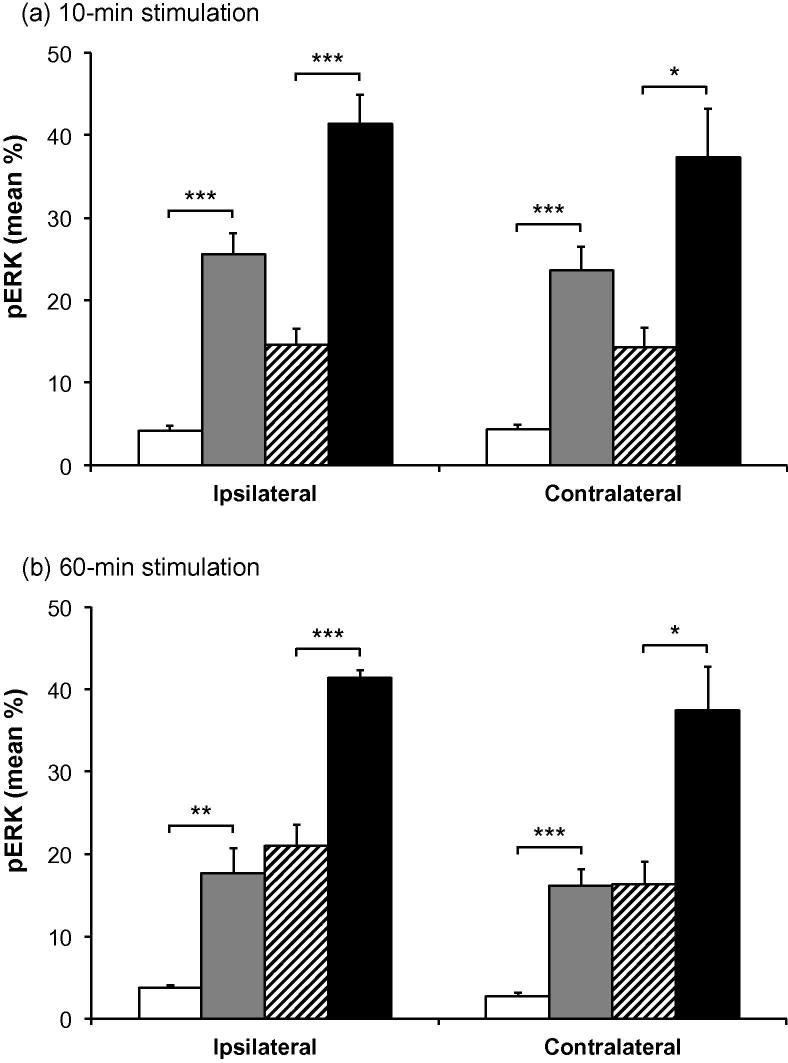
Mean pERK immunoreactivity in subnucleus caudalis. Mean percentage area (±SEM) of pERK labeling in subnucleus caudalis of animals with non-inflamed or inflamed tooth pulps, either non-stimulated or stimulated for (a) 10 min or (b) 60 min. □ = non-stimulated, non-inflamed;  = non-stimulated, inflamed;  = stimulated, non-inflamed; ■ = stimulated, inflamed. The graphs show both ipsilateral and contralateral sides. Each bar represents the average percentage area staining of seven sections in the area 1800–1080 μm caudal to obex. ^∗∗∗^*p* < 0.001; ^∗∗^*p* < 0.01; ^∗^*p* < 0.05; unpaired *t*-test.

**Fig. 6:**
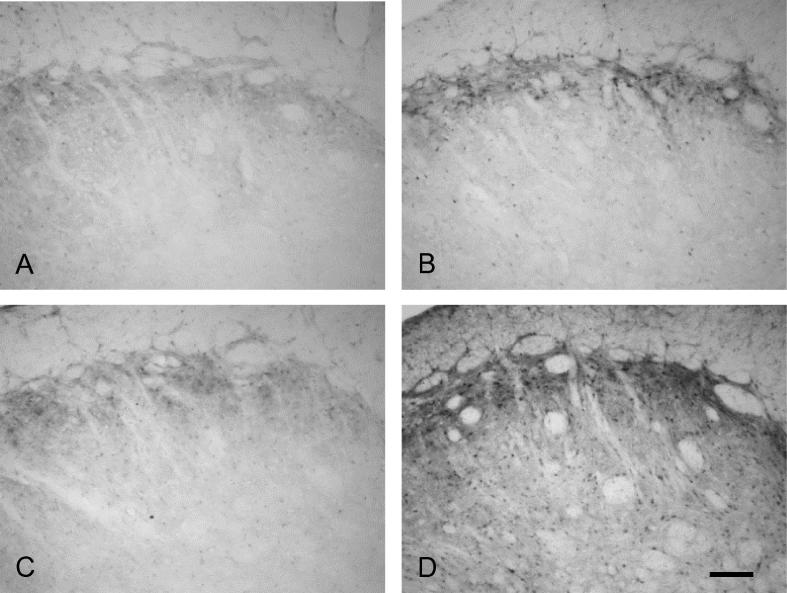
Examples of pp38 immunoreactivity in subnucleus caudalis. Photomicrographs showing examples of ipsilateral pp38 labeling in subnucleus caudalis (1800 μm caudal to obex). (A) non-stimulated, non-inflamed; (B) non-stimulated, inflamed; (C) stimulated (60 min), non-inflamed; (D) stimulated (60 min), inflamed. Scale bar = 100 μm.

**Fig. 7:**
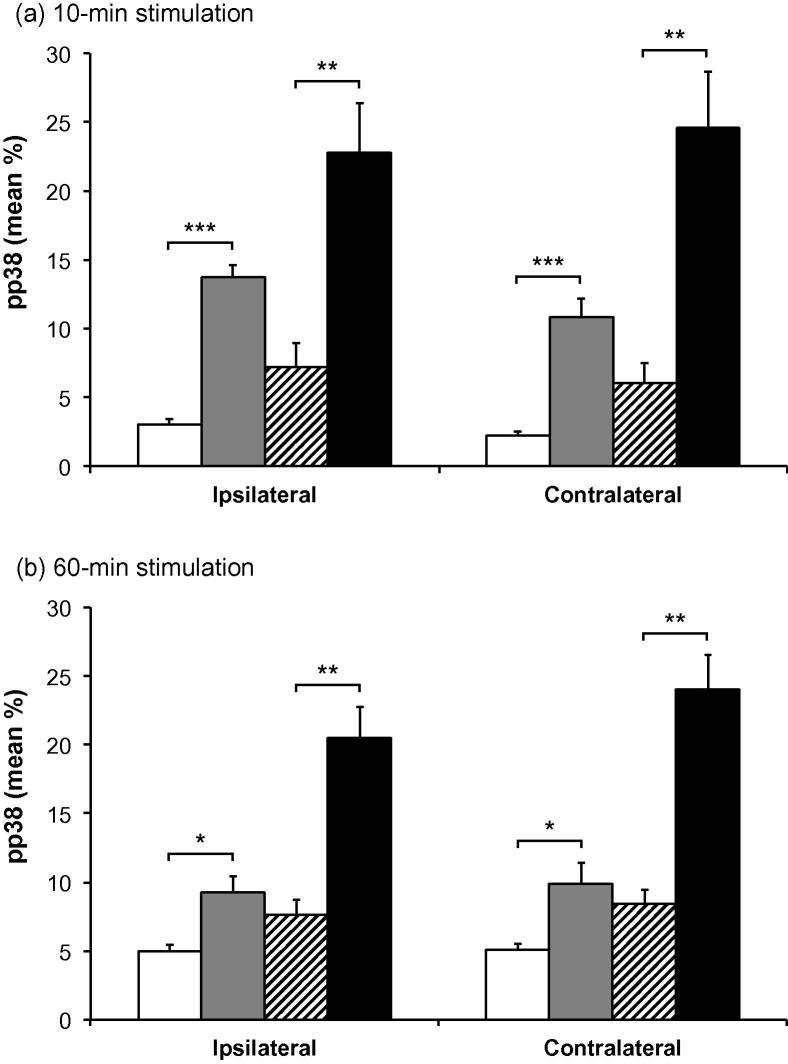
Mean pp38 immunoreactivity in subnucleus caudalis. Mean percentage area (±SEM) of pp38 labeling in subnucleus caudalis of animals with non-inflamed or inflamed tooth pulps, either non-stimulated or stimulated for (a) 10 min or (b) 60 min. □ = non-stimulated, non-inflamed;  = non-stimulated, inflamed;  = stimulated, non-inflamed; ■ = stimulated, inflamed. The graphs show both ipsilateral and contralateral sides. Each bar represents the average percentage area staining of seven sections in the area 1800–1080 μm caudal to obex. ^∗∗∗^*p* < 0.001; ^∗∗^*p* < 0.01; ^∗^*p* < 0.05; unpaired *t*-test.

Figs. 5 and 7 show the mean percentage areas of pERK and pp38 expression in Vc. Percentage area of expression for different groups was similar at both 10- and 60-min time points. Mean percentage expression of pERK and pp38 was lowest in non-stimulated non-inflamed animals and highest in stimulated inflamed animals, with the other two groups having an intermediate degree of labeling.

A two-way ANOVA demonstrated a highly significant effect of inflammation (*p* < 0.0001 in all cases), and further pairwise comparisons showed a significant effect of inflammation in both non-stimulated and stimulated groups (*p* < 0.05 in all cases, unpaired *t*-test; Figs. 5 and 7). Two-way ANOVA also identified an additional but less powerful effect of stimulation (*p* ⩽ 0.0072 in all cases).

### Double labeling

Some sections were utilized to examine colocalization of pERK and pp38 with NeuN to label neurons and GFAP to label astrocytes. Attempts were also made to investigate expression in microglia by labeling for OX42 and Iba-1, however neither of these antibodies produced any clear labeling. Both of these markers have been widely used in rat and mouse but to the authors’ knowledge there are no reports of their use in ferret. It seems likely that the lack of positive staining in this study was related to species differences in microglial cells.

**Fig. 8:**
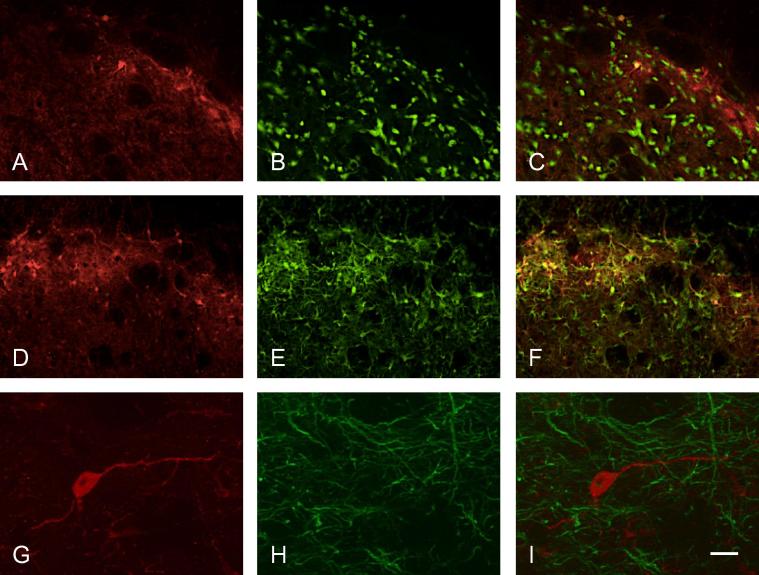
Dual labeling of pERK with NeuN and GFAP. Dual labeling of pERK (red) with (A–C) NeuN or (D–I) GFAP (both green); co-localization appears yellow. Sections in (A–C) and (G–I) stimulated only; section in (D–F) stimulated, inflamed. (G–I) shows a higher power photomicrograph of a single pERK-labeled neuron. Scale bar = 100 μm (A–F); 25 μm (G–I).

**Fig. 9:**
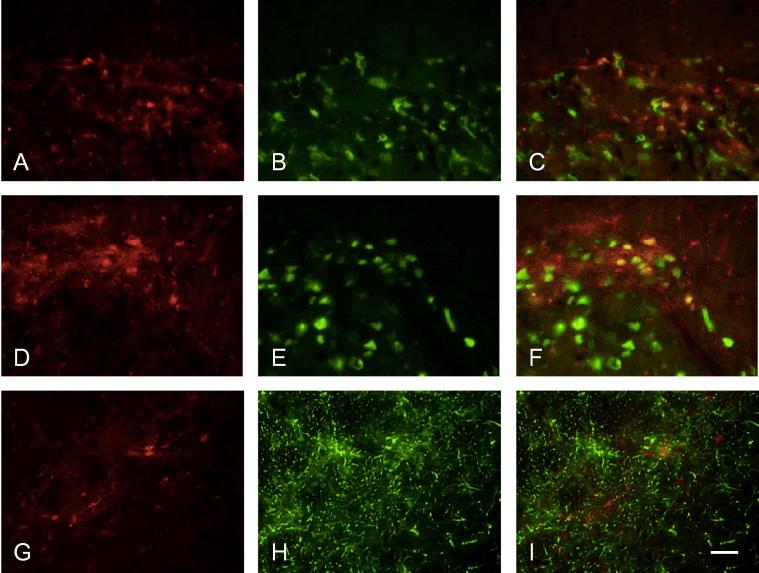
Dual labelling of pp38 with NeuN and GFAP. Dual labeling of pp38 (red) with (A–F) NeuN or (G–I) GFAP (both green); co-localization appears yellow. Section in (A–C) inflamed only; section in (D–F) stimulated, inflamed; section in (G–I) stimulated only. Scale bar = 50 μm.

Labeling for pERK was colocalized with NeuN in animals that had been stimulated (Figs. 8A–C). However in animals with inflamed pulps, a large proportion of pERK labeling was colocalized with GFAP (Figs. 8D–F). pERK-positive cells with clear neuronal profiles that did not express GFAP were also seen (Figs. 8G–I).pp38 was also found to be present in neurons (Figs. 9A–F); qualitatively, there appeared to be more labeled neurons in animals with stimulated inflamed pulps (Figs. 9D–F). Labeling for pp38 was also present in other cells and terminals not labeled by NeuN, and no colocalization was seen with GFAP (Figs. 9G–I) following either inflammation or stimulation. This suggests that labeling not colocalized with NeuN was in microglial cells.

## Discussion

In this study we have compared the expression of Fos, pERK and pp38 following chronic tooth pulp inflammation and acute stimulation. As demonstrated in our previous work (Worsley et al., 2007), the study showed that chronic inflammation of the pulp alone did not induce Fos expression, but Fos expression was increased following stimulation in animals with inflamed teeth. However, the expression of both pERK and pp38 displayed a different pattern to that of Fos. Chronic inflammation alone or stimulation alone increased bilateral expression of these molecules in Vc, and expression was further increased in animals that were both stimulated and inflamed. The lack of Fos expression following stimulation alone is in contrast to previous studies that have used inflammatory stimuli to evoke Fos expression in the trigeminal complex. Inflammation of the dental pulp (Byers et al., 2000; Chattipakorn et al., 2002) or temporomandibular joint (Zhou et al., 1999) has been shown to increase Fos expression bilaterally in Vc and the transitional regions between subnuclei caudalis and interpolaris. As discussed in detail in our previous study (Worsley et al., 2007), these differences may be explained by the use of different protocols, species or models.

### Time course of pERK and pp38 expression

Our present study demonstrates for the first time that unlike Fos, both pERK and pp38 are present 6 days following induction of chronic pulpal inflammation, even in the absence of further pulpal stimulation. This indicates that chronic pulpal inflammation produces altered central activation, which is likely to be linked to the increased Fos expression seen in animals with inflamed stimulated pulps, compared with that seen in those with non-inflamed stimulated pulps. It has been clearly established that a wide range of peripheral stimuli induce activation of ERK and p38 in the dorsal horn and trigeminal nucleus (Ji et al., 2009). This includes studies of pERK expression following the application of capsaicin to the tooth pulp (Shimizu et al., 2006); however this previous study showed only very transient activation of pERK that was maximal at 5 min and back to baseline levels at 2 h following stimulation. It may not be appropriate to draw comparisons between the time course of ERK activation in this and the current study as capsaicin application is an acute stimulus, but it does highlight major differences in longevity of altered central signaling in a range of models of inflammatory and neuropathic pain.

Other studies have also indicated that following induction of inflammation, expression of pERK and pp38 is relatively short lived, even following application of stimuli that induce relatively long-term inflammatory changes. For example, following injection of CFA into the hind paw, pERK is only transiently expressed in the dorsal horn (peaking at 10 min and reverting to baseline levels by 2 days), despite the presence of on-going peripheral inflammation, heat hyperalgesia and mechanical allodynia (Ji et al., 1999; Gao and Ji, 2010). However, in a number of studies of inflammatory pain, pERK expression has been shown to be increased for longer time periods post-induction of inflammation. For example, a study in the trigeminal system examined pERK expression following injection of CFA into the tongue, and reported that levels were still elevated 8 days following induction of inflammation (Liu et al., 2012).

Many studies of p38 following inflammation show similar, short-lived activation of this molecule. However some models of inflammation or injury do induce longer-term expression of pp38 in the spinal cord, including burn injury (up to 4 weeks) (Chang and Waxman, 2010) and adjuvant-induced arthritis, where pp38 is expressed between 8 and 17 days (Boyle et al., 2006). A further study of formalin injury has shown that pp38 is expressed in two stages, the first appeared a few minutes after injection and peaked at 1 h and the second was maximal 3–7 days after injury (Li et al., 2010). In the trigeminal system, injection of CFA into the temporomandibular joint induces expression of pp38 that still persists at 7 days post injection (Cady et al., 2010).

It is also well established that expression of pERK and pp38 is increased in the dorsal horn and trigeminal nucleus following peripheral nerve injury, where persistent expression has been reported in many studies. Zhuang and colleagues investigated the time course of pERK expression following a spinal nerve ligation injury (Zhuang et al., 2005). Their study showed that following this injury, pERK levels are rapidly elevated (within 10 min) and are still raised 3 weeks post injury. However, in the trigeminal system, ERK activation following nerve injury appears less persistent with reports of maximal expression ranging from 1 day (Terayama et al., 2011) to 3 days (Shibuta et al., 2012) following injury. Persistent activation of p38 (detectable at day 1, maintained for 3 weeks) is known to occur in the spinal cord following nerve injury (Jin et al., 2003), and in the trigeminal nucleus, raised levels of pp38 have also been reported to be present and to persist for 3–14 days post nerve injury (Piao et al., 2006; Lee et al., 2011; Terayama et al., 2011; Daigo et al., 2012).

Thus the time course of expression of these molecules is highly dependent on the nature of the stimulus, and this has significant implications in terms of how data from these studies might relate to clinical pain in man. In general, models with close clinical correlates – including the one used in the present study – appear to show more sustained elevation of these molecules than those using acute stimulation. For example, models of cancer pain have demonstrated sustained elevation of pERK and pp38, with elevated pERK persisting for 21 days post intra-tibial inoculation with cancer cells (Wang et al., 2012), and elevated pp38 still present at 14 days post injection of osteosarcoma cells into the femur (Svensson et al., 2008). Thus it may be that data from some preclinical models may underestimate the time course of activation and the potential role of these molecules in pain states in man.

Other stimuli have clear differential effects on the time course of activation of ERK and p38, as demonstrated in a study examining expression of pERK and pp38 in a model of bee venom-induced inflammation and hyperalgesia (Cui et al., 2008). This stimulus (intraplantar injection of bee venom) induced ERK activation, which peaked at 2 min but had returned to baseline levels by 48 h; whereas p38 activation first appeared at 1 h, peaked at 3 days and was still present 7 days after stimulation.

### Bilateral expression of pERK and pp38

In the present study both pERK and pp38 were present bilaterally and levels of expression were similar in both ipsi- and contralateral Vc, whereas Fos expression was only present ipsilateral to pulpal stimulation. Previous studies of acute pulpal stimulation with capsaicin also report bilateral expression of pERK in Vc (Shimizu et al., 2006); however in contrast to the present study, these authors also report bilateral Fos expression following this stimulus. Bilateral expression of pERK and pp38 is not generally seen in the spinal cord, but has been reported in response to some stimuli, including burn injury (Chang and Waxman, 2010) and in a model of cancer-induced pain (Wang et al., 2012). It has been postulated that these contralateral changes are linked to the development of contralateral allodynia. A recent study of over 2000 patients with referred dental pain reported that 15% of these patients had pain referred to the opposite side (Hashemipour and Borna, 2014). It is plausible that contralateral changes in pERK and pp38 expression may be involved in such contralateral changes in sensitivity in patients with pulpal inflammation.

### Colocalization with NeuN and GFAP

This study did not quantify colocalization of pERK and pp38 in different cell types, however some interesting patterns of colocalization were observed. We showed pERK to be present in both neurons and astrocytes. Many studies have shown that pERK is expressed in neurons following application of a range of acute stimuli (Ji et al., 2009), and the current study found pERK expression in neurons especially following electrical stimulation. However our study also showed that pERK was expressed in astrocytes, and this was particularly common in animals with inflamed tooth pulps. Expression has previously been described in astrocytes in some other models of inflammation and injury. Relatively recent studies in models of cancer-induced bone pain demonstrated that pERK was predominantly expressed in astrocytes at 12 and 14 days after inoculation of bone with tumor cells (Wang et al., 2011, 2012). A previous study of nerve injury by Zhuang et al. (2005) also showed activation of ERK at longer time periods after injury, with sequential activation in neurons, microglia and astrocytes. The authors report that following injury, pERK in neurons and microglia peaked at 10 min and 2 days, respectively; at 10 days, expression was equally present in microglia and astrocytes, and by 21 days the majority of labeling was present in astrocytes. In the trigeminal nucleus, pERK has also been shown to be present in astrocytes following lingual nerve injury (Terayama et al., 2011); as in the study by Zhuang et al. (2005), this study showed a progression of pERK expression which was seen mostly in microglia at 1 and 3 days and in astrocytes at 7 days. A role for astrocytes in orofacial hyperalgesia is also supported by pharmacological studies of chronic pulpitis and nerve injury-induced pain in the trigeminal system, which have shown that modulation of astrocyte activity at 7–14 days post injury is effective in reversing behavioral responses and electrophysiological activity indicative of hyperalgesia and central sensitization (Okada-Ogawa et al., 2009; Lee et al., 2011; Tsuboi et al., 2011).

Our present study also demonstrated that pp38 was expressed in neurons, but we did not see any expression in astrocytes. We were not able to examine pp38 expression in microglia, since – as reported in the results – neither Iba-1 nor OX42 labeling was successful. Many studies have reported p38 activation in microglia, especially following acute stimulation (Wen et al., 2011), however there are also some previous studies showing pp38 expression in neurons. This has been reported in a number of models of pain including postoperative pain (Huang et al., 2011), adjuvant-induced arthritis (Boyle et al., 2006), cancer pain (Svensson et al., 2008) and temporomandibular joint inflammation (Cady et al., 2010). These studies using chronic pain models have reported pp38 in neurons at relatively late time periods, ranging from 7 to 14 days. A further study has reported pp38 expression in neurons following bee venom injection (Cui et al., 2008), and this appeared as early as 1 day after injection and still persisted 7 days after injection.

Together, these studies suggest that sequential activation of ERK and p38 in different cell types may be a common feature of disease progression. This may be highly relevant in translating data from preclinical models to chronic pain conditions in man.

### Clinical correlates

It is well documented that patients with pulpitis report altered sensitivity of the affected teeth. This may present as spontaneous pain or increased sensitivity, particularly in response to thermal stimuli. Our group has developed precise methods to quantify pulpal sensitivity to thermal stimuli and recent studies have demonstrated a clear correlation between the presence of pulpal inflammation and increased thermal sensitivity in man (Barber et al., 2012). Studies from this laboratory have also shown significant correlations between clinical pain history and altered expression of a variety of molecules within the tooth pulp (Rodd and Boissonade, 2000, 2001; Morgan et al., 2005, 2009). The present study indicates that as well as these peripheral changes, long-term central changes in MAPK signaling are present under conditions of chronic pulpal inflammation and both are likely to play a significant role in the generation of clinical pain symptoms.

## Conclusions

This study provides the first demonstration that chronic inflammation of the tooth pulp induces significant and persistent bilateral activation of pERK and pp38 within the trigeminal nucleus, and that expression of these molecules is further increased by acute stimulation. This altered activity in intracellular signaling is likely to be linked to the increased Fos expression that is seen in the inflamed stimulated animals in this and our previous studies, and is likely to be relevant to the increased sensitivity that is seen in patients with pulpitis. Data from this study also indicate that pERK and pp38 are more accurate markers of central change than Fos expression. In this model of chronic inflammation, colocalization of pERK and pp38 within specific cell types differs from that reported following the application of acute stimulation. These findings provide further evidence for sequential activation of these molecules in different cell types in models of chronic pain, and may indicate specific roles for different cell types in induction and maintenance of pulpitic and other types of pain.

## Role of the funding source

The BBSRC together with GlaxoSmithKline funded an Industrial Partnership Award to FMB and AEK. GlaxoSmithKline were involved in project progress meetings and have approved the submission of the manuscript.

## Competing interests

There were no competing interests.

## Authorship

FMB with AEK conceived and supervised the study, and FMB drafted the manuscript. MAW prepared the animals, analyzed the data and carried out the immunohistochemistry with assistance from CEA. AB was industrial supervisor.

## Figures and Tables

**Table 1:** Primary and secondary antisera

	Host and type	Dilution	Supplier
Antigen			
Fos	Rabbit polyclonal	1:20,000	Calbiochem, USA
pERK	Rabbit polyclonal [#9101]	1:600	Cell Signaling Technology, USA
pp38	Rabbit polyclonal [#9211]	1:300	Cell Signaling Technology, USA
NeuN	Mouse monoclonal	1:500	Abcam, UK
GFAP	Mouse monoclonal	1:300	Vector Laboratories, UK

Secondary label			
Biotin	Goat anti-rabbit	1:300	Vector Laboratories, UK
Cy3	Donkey anti-rabbit	1:500	Jackson ImmunoResearch, UK
FITC	Donkey anti-mouse	1:500	Jackson ImmunoResearch, UK
